# Policies beyond the crisis: lesson learned

**DOI:** 10.1186/2052-3211-8-S1-E2

**Published:** 2015-10-05

**Authors:** Sabine Vogler, Nina Zimmermann, Veronika J Wirtz, Zaheer-Ud-Din Babar

**Affiliations:** 1 WHO Collaborating Centre for Pharmaceutical Pricing and Reimbursement Policies, Health Economics Department, Gesundheit Österreich GmbH (Austrian Public Health Institute), Vienna, 1010, Austria; 2 Department of Global Health, Boston University School of Public Health, Boston, MA 02118, USA; 3 School of Pharmacy, Faculty of Medical and Health Sciences, University of Auckland, Private Mail Bag 92019, Auckland, New Zealand

## 

Some European and further countries have been hit by the global financial crisis since 2008, and some of them were hit hard. The crisis has had major impacts on the health systems, including the pharmaceutical sector since cost-containment measures with possible negative impacts were set.

## Responses to the global financial crisis

According to a survey with the PPRI (Pharmaceutical Pricing and Reimbursement Information) network (see also E4), a total of 445 measures (related to pharmaceutical pricing and reimbursement) were reported during the period of 2010-2014. This corresponds to, on average, nearly 13 measures per country, but with great variability between the countries (range: 2-44). The most frequently reported policy measures were price cuts, followed by changes in co-payment and in the reimbursement lists (formularies). The highest number of measures (130 measures) was reported for 2012 in which the crisis was at the peak in some countries. Overall, during the 4 years, Portugal was the country that reported the highest number of measures, followed by Belgium, France and Iceland. An analysis for merely 2010-2011 evidenced that price cuts and changes in co-payments were also the policy measures taken most frequently in that time period. But countries with the highest number of measures were different at that time; this included Iceland, the Baltic States (Estonia, Latvia, Lithuania), Greece, Spain and Portugal [1].

Despite the limitations of that survey (in particular a possible reporting bias), the study suggested that during the global financial crisis a higher number of pharmaceutical policy measures were taken, several had a focus on cost-containment, and that these were frequently measures that could be undertaken short term. However, a crisis might also offer an opportunity to move forward with policy options that had not been feasible at other times. Despite this focus on cost-containment during the crisis, policies to achieve other objectives, such as encouraging a more effective and efficient use of medicines and promotion of generic policies, could also be observed, in the Baltic states, for instance [2-4].

Cuts in public expenses risk negatively impacting health outcomes. First analyses available for Greece suggest that the crisis, and the policy responses to it, brought negative effects on the quality of health services, a lower utilization of health care and signs of worse health outcomes, such as increasing rates of mental health, suicides, and epidemics, and a deterioration of self-rated health [5,6].

There is less evidence on the impact of the crisis in the pharmaceutical sector. The effect on public pharmaceutical expenditure was observed, with modest and even negative growth rates in the ‘crisis countries’ during some years [7,8]. Vandoros and Stargardt [9] concluded for Greece that despite a major drop in pharmaceutical expenditure, more cuts would be necessary and could only be achieved through increased efficiency if the quality of healthcare and public health should not be compromised. They also addressed the threat that companies could withdraw from the Greek market in the light of the low price levels for originator medicines to which several other European countries refer to in their price setting [10]. However, product withdrawals took place at a very small scale in Greece [9].

More research is needed to learn whether, or not, access to medicines has been negatively impacted by the crisis. A study on eight European countries (three economically stable, and five less economically stable countries) showed that although less economically stable countries implemented more pharmaceutical policy changes during the recession than economically stable countries, pharmaceutical sales volumes (quarterly sales of products in the 10 highest-selling therapeutic classes in each country between 2006 and 2011) increased in almost all countries, whereas sales values declined, especially in less stable countries [11]. The authors suggested that the decline in the value of sales with the increases in volume might indicate that pharmaceutical purchasing had become more efficient. However, an analysis focusing on only two countries (Finland and Portugal) and one group of medicines (antipsychotics) showed slight, probably unintended, decreases in overall use of antipsychotic medicines and increases in generic market shares of major antipsychotic products from January 2007 to December 2011 [12].

## Policy approaches in ‘non-crisis’ countries

Strand 2 at the 2015 Vienna PPRI Conference explores which pharmaceutical policies, particularly in the area of pharmaceutical pricing and/or reimbursement, countries chose to respond to the financial crisis. A key note by Panos Kanavos (London School of Economics) addresses the situation in Greece. Susan Spillane (National Centre for Pharmacoeconomics, Ireland, O10) looks at the impact of generic substitution and the reference price system that were recently introduced in Ireland, on pharmaceutical expenditure and generic penetration. Susanne Mayer (Medical University of Vienna, O12) analyses whether, or not, socioeconomic inequalities in medicine use exist in Central Eastern Europe, and how pharmaceutical policies can reduce them.

However, the PPRI Conference will not only focus on the countries hit by the financial crisis. It is also of interest which policy options were implemented and/or are being discussed in ‘non-crisis’ countries, and which have been the impacts of such policies. Countries in Europe and beyond are investigated. A key-note by Zaheer Ud-Din Babar (University of Auckland, K5) will inform about pharmaceutical policies in Australia and New Zealand. With regard to low- and middle-income countries, Yared Santa-Ana-Tellez (Utrecht University, O11) analyses price changes of antibiotics and perceived substitutes in the context of policy changes related to Over-the-Counter medicines in Mexico and Brazil. Peter Schneider (Austrian Public Health Institute, O4) analyses the impact of discounts on the medicine price levels.

In a country poster session numerous pharmaceutical systems from Europe and the world over will be presented.
